# Minimally invasive pedicle screw fixation utilizing O-arm fluoroscopy with computer-assisted navigation: Feasibility, technique, and preliminary results

**DOI:** 10.4103/2152-7806.68705

**Published:** 2010-08-25

**Authors:** Paul Park, Kevin T. Foley, John A. Cowan, Frank La Marca

**Affiliations:** Department of Neurosurgery, University of Michigan Health System, Ann Arbor, MI, USA; 1 Department of Neurosurgery, University of Tennessee Health Science Center, Memphis, TN, USA

**Keywords:** Image-guided surgery, minimally invasive procedures, O-arm, pedicle screw

## Abstract

**Background:**

Pedicle screw misplacement is relatively common, with reported rates ranging up to 42%. Although computer-assisted image guidance (CaIG) has been shown to improve accuracy in open spinal surgery, its use in minimally invasive procedures has not been as well evaluated. We present our technique and review the results from a cohort of patients who underwent minimally invasive lumbar pedicle screw placement utilizing the O-arm imaging unit in conjunction with the StealthStation Treon System.

**Methods:**

A retrospective review of patients who underwent minimally invasive pedicle screw fixation with CaIG was performed. Eleven consecutive patients were identified and all were included. Nine patients underwent a single-level transforaminal lumbar interbody fusion. Two patients underwent multi-level fusion. Inaccurate pedicle screw placement was determined by postoperative computed tomography (CT) and graded as 0–2, 2–4, 4–6, or 6–8 mm.

**Results:**

A total of 52 screws were placed. Forty screws were inserted in eight patients who had postoperative CT, and a misplacement rate of 7.5% was noted including one lateral and two medial breaches. All breaches were graded as 0–2 mm and were asymptomatic. In the remaining three patients, post-instrumentation O-arm imaging did not demonstrate pedicle screw misplacement.

**Conclusion:**

Although this initial study evaluates a relatively small number of patients, minimally invasive pedicle screw fixation utilizing the O-arm and StealthStation for CaIG appears to be safe and accurate.

## INTRODUCTION

Pedicle screw misplacement is a potential complication of instrumented spinal fusion, which can lead to neurologic injury.[1,2] In this study, we evaluate a cohort of patients who underwent minimally invasive lumbar spinal fusion utilizing the O-arm^™^ Imaging System (Breakaway Imaging, Littleton, MA, USA) with computer-assisted image guidance (CaIG) for placement of pedicle screws. The O-arm is a recent innovation that combines intraoperative fluoroscopy with the capability of multi-dimensional imaging. Its ability to obtain computed tomography (CT)-type images with multi-planar reconstructions and an automated registration to the StealthStation Treon Guidance System (Medtronic, Minneapolis, MN, USA) makes it ideally suitable for minimally invasive procedures. To date, no study has evaluated the O-arm for minimally invasive pedicle screw insertion. The operative technique and results are reviewed.

## METHODS

After getting approval from the institutional review board, a retrospective review of computerized medical records was performed. From December 2007 to April 2008, 11 consecutive patients underwent minimally invasive lumbar pedicle screw fixation utilizing the O-arm with Stealth navigation. All the patients were included in the study. Demographic information, surgical data, radiographic data, and complications were recorded.

Nine patients underwent a single-level minimally invasive transforaminal lumbar interbody fusion (MI-TLIF) [Table 1]. A “hybrid” procedure combining minimally invasive and mini-open components was performed in the remaining two patients.

**Table 1:** Demographics, diagnosis, and surgical procedures in patients receiving minimally invasive pedicle screw placement

Number of patients	11
Mean age (years)	44.0 (range 19–62)
Sex
Male	7 (63.6%)
Female	4 (36.4%)
Diagnosis
Degenerative disk disease	3 (27.3%)
Degenerative spondylolisthesis	2 (18.2%)
Isthmic spondylolisthesis	4 (36.3%)
Degenerative scoliosis and spondylolisthesis	1 (9.1%)
Idiopathic scoliosis	1 (9.1%)
Procedure
L4–5 MI-TLIF	6 (54.5%)
L5–S1 MI-TLIF	3 (27.3%)
MI L2–5 fixation, mini-open laminectomy	1 (9.1%)
L1–L4 MI-TLIF, open thoracic osteotomies/fixation	1 (9.1%)

MI-TLIF = minimally invasive transforaminal lumbar interbody fusion

### Surgical technique

For the MI-TLIF, either the Sextant (Medtronic Sofamor Danek, Minneapolis, MN, USA) or the Viper (Depuy Spine, Raynham, MA, USA) system was used for pedicle screw fixation. Access for performing the decompression and interbody cage placement was obtained using the METRx tubular retraction system (Medtronic Sofamor Danek). The StealthStation Treon Guidance System with Synergy software (Medtronic) was used for CaIG, in conjunction with the O-arm fluoroscopic unit.

**Figure 1:**
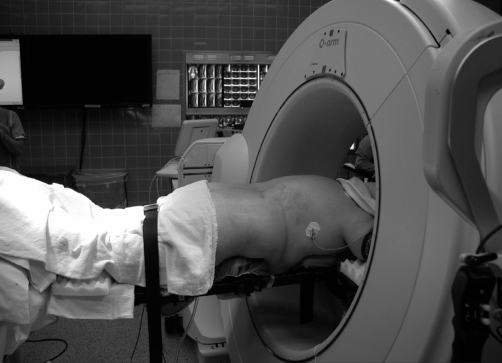
Typical patient positioning with the O-arm in the “parked” position

Each patient was positioned prone on a radiolucent Jackson frame with the StealthStation camera placed at the foot. The O-arm was then positioned so that the target spinal segment was centered in the field of view. To accomplish this, anterior-posterior (A/P) and lateral views were obtained in 2-D fluoroscopy mode. The O-arm was then moved toward the patient’s head to the “parked” position, which allowed access to the patient for the surgical procedure [Figure 1]. After standard skin sterilization and draping, the O-arm was re-positioned so that the target spinal segment was centered within the O-arm ring. A stab incision was made over the posterior iliac crest and a percutaneous iliac pin was placed. The StealthStation reference arc was then attached to the iliac pin. At this point, the O-arm in 3-D multi-planar mode was used to obtain a CT-type image of the spine. The O-arm then transferred the image data to the StealthStation for auto-registration and production of multiplanar images that included trajectory viewpoints. At this point, image guidance was ready for use. Of note, no surgeon-derived registration of the spine to the StealthStation 3-D image was necessary.

**Figure 2:**
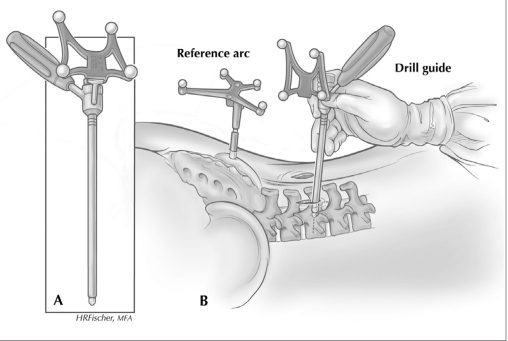
Diagram showing the technique for K-wire insertion through pedicle, using LDG with reference arc positioned toward the StealthStation camera at the foot of the operating room table

**Figure 3:**
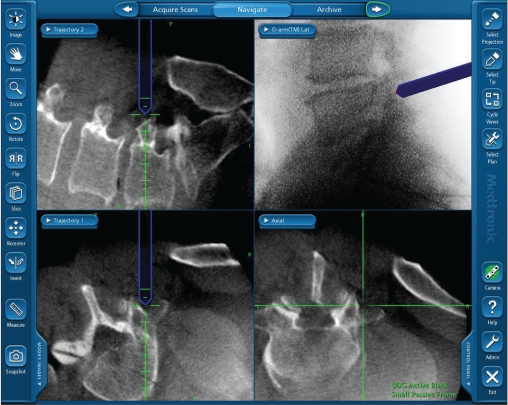
Typical image from StealthStation during image guidance showing trajectory views of LDG

With the O-arm moved to the patient’s head, two paramedian 1-inch incisions overlying the target spinal segment were then made. The Stealth long drill guide (LDG) was then inserted through the fascia and used to locate the proper starting site and trajectory for pedicle screw placement. Positioning of the LDG was entirely based on image guidance without visualization of the spinal anatomy [Figures 2 and 3]. After appropriate positioning, a K-wire was placed through the LDG and driven into the pedicle to an approximate depth of 3 cm. This process was repeated for the remaining pedicles. A/P and lateral fluoroscopic views were obtained after placement of all the K-wires to confirm adequate placement. The K-wires were then gently splayed to either side and anchored with a snap. From the most symptomatic side, a tubular retractor was then inserted over the target disk space, using one of the existing incisions. This was followed by TLIF, performed in a manner similar to that described by Holly *et al*.[4] After the TLIF, a pedicle screw connected to a screw extender was inserted over each K-wire into the pedicle. The screw extenders were then used to align the screw heads so that a rod could be placed. After placement of the set screws, the screw extenders were removed.

### Radiographic analysis

Postoperative imaging in eight patients consisted of a standard spine protocol CT scan with 2.5 mm slices. In the remaining three patients (4, 6, and 11), the O-arm was utilized to confirm adequate screw placement, so postoperative CT was not performed. Patient 6 did, however, undergo postoperative, non-spine protocol CT.

Accuracy of screw placement was recorded using a previously reported scale in which medial or lateral penetration of the cortex was graded as 0–2, 2–4, 4–6, or 6–8 mm.[1,8]

## RESULTS

**Figure 4:**
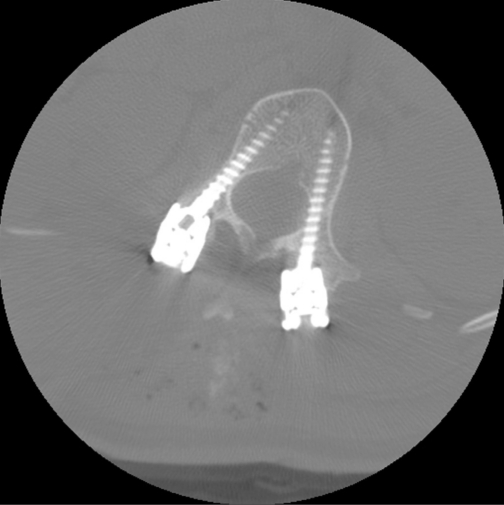
Axial postoperative CT showing medial breach of L1 pedicle. Note the significant rotational component to the patient’s scoliosis

There were seven male patients (63.6%) in our study. The mean age for all patients was 44.0 years (range, 19–62 years). A total of 52 screws were placed. In the eight patients who had postoperative spine protocol CT imaging with sagittal and coronal reformats, 40 screws were placed. There were three (7.5%) instances out of the 40 screw insertions where the cortex was penetrated, consisting of two medial breaches and one lateral breach. All three cases were graded as 0–2 mm and none were symptomatic [Figure 4].

**Figure 5:**
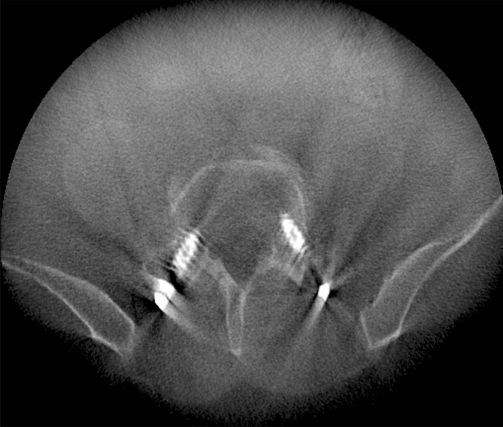
Axial image from the O-arm showing adequate positioning of pedicle screws

Twelve additional pedicle screws were placed in three patients who did not undergo postoperative spine protocol CT imaging. One patient had a non-spine protocol CT, which upon review, did not show evidence of screw misplacement. In the remaining two patients, the O-arm was used to check screw placement at the end of the procedure. Although not of the same diagnostic quality as a true CT scanner, the O-arm images obtained did not demonstrate significant medial or lateral screw misplacement [Figure 5]. None of these three patients had clinical evidence of a radiculopathy.

## DISCUSSION

Even with fluoroscopic assistance, inaccurate pedicle screw placement can be relatively common and can result in neurologic injury. Castro *et al*.[1] evaluated 30 consecutive patients who underwent a total of 123 pedicle screw placements by experienced spine surgeons, using fluoroscopy. On postoperative CT imaging, 49 (40%) screws had penetrated either the medial or lateral cortex of the pedicle. Of the 35 (29%) screws that had a medial breach, 22 were 0–2 mm, 4 were 2–4 mm, 2 were 4–6 mm, and 7 were 6–8 mm. Symptomatic neurologic injury resulted in five patients, all of whom had screws intruding 6-8 mm. Subsequent studies have shown similar results, with pedicle screw misplacement rates of 21.0 and 28.1%.[3,6]

In comparison to fluoroscopy-assisted screw placement, CaIG has been shown to achieve overall higher rates of accuracy. Merloz *et al*.[7] performed a comparative study evaluating 52 screws placed with conventional techniques and 52 screws placed with CaIG, and noted the misplacement rates to be 42 and 8%, respectively. Similarly, in a randomized controlled trial of 50 patients undergoing fluoroscopy-assisted screw insertion compared to 41 patients undergoing screw insertion with CaIG, the rates of misplacement were 13.4 and 4.6%, respectively.[5] Although 1.4% of the screws in the fluoroscopy-assisted group had intrusion of 4–6 mm, there were no breaches greater than 4 mm in the CaIG group.

Although many studies have now shown CaIG to increase accuracy in open spinal procedures, the use of CaIG in minimally invasive cases has not been as well evaluated. In minimally invasive procedures, exposure of the spine is typically very limited. Since early versions of CaIG relied on preoperative CT images of the spine registered to the patient intraoperatively by means of a point registration to the exposed spine, the lack of bony exposure limited the use of CaIG. However, a relatively recent innovation, the Siremobil IsoC 3-D fluoroscope (Siemens Medical Solutions, Erlangen, Germany), has made the use of CaIG in minimally invasive procedures both practical and feasible. Instead of relying on a preoperative CT image, the Iso-C can acquire multi-planar images intraoperatively with the patient positioned for surgery. After positioning of the StealthStation camera and placement of the reference arc, the Iso-C fluoroscope obtains successive images while rotating 190° around the patient. Approximately three adjacent vertebral levels can be imaged. This data are then transferred to the StealthStation navigation system, which performs an auto-registration process. Since there is no requirement for surgeon-derived point registration to the exposed spine, the use of Iso-C for minimally invasive pedicle screw fixation is possible. Villavicencio *et al*. [8] reviewed a series of 69 patients who underwent instrumentation assisted with the Iso-C and CaIG. Forty-six of the patients underwent lumbar fusion consisting of either a single- or 2-level TLIF with percutaneous pedicle screw fixation. A total of 220 screws were inserted, and 4 (1.8%) were considered misplaced. Breaches of 0–2 mm in two cases and 4–6 mm in two cases were noted, which necessitated re-positioning.

The O-arm fluoroscopic unit is a new imaging device that incorporates a flat panel detector and the X-ray source in a cylindrical bore. The cylindrical bore can be opened laterally to allow positioning around the operating room table. The O-arm can acquire standard fluoroscopic images as well as multi-planar images of the spine. Image acquisition in multi-planar mode involves successive images obtained while the X-ray source and detector rotate 360° around the patient. Similar to the Iso-C, data are automatically transferred to the StealthStation where auto-registration occurs for CaIG. Purported advantages of the O-arm compared to the Iso-C include improved multi-planar image quality due to the larger number of images obtained during the 360° acquisition, larger field of view, and robotic re-positioning of the O-arm to pre-programmed positions facilitating subsequent fluoroscopic views. One disadvantage is the expense of the O-arm fluoroscopic unit, which currently lists for approximately $700,000.00. In addition, the StealthStation Treon Guidance System costs approximately $250,000.00 [Table 2].

**Table 2:** Comparison of image guidance options for pedicle screw placement

	Fluoroscopy-assisted	Preoperative CT-based CaIG	Intraoperative Iso-C-based CaIG	Intraoperative O-arm-based CaIG
Advantages	↓Time	↑Accuracy	↑Accuracy	↑Accuracy*
	↓Cost	↓Surgeon radiation exposure	↓Surgeon radiation exposure	↓Surgeon radiation exposure
			↓Time (versus preoperative CT- based CaIG)	↓Time (versus preoperative CT-based CaIG)
			Can acquire intraoperative multi- planar images	Can acquire intraoperative multi-planar images
			Can act as a fluoroscope	Can act as a fluoroscope
				↑Image quality (versus Iso-C-based CaIG)**
				↑Field of view (more spinal segments can be imaged)**
				Robotic re-positioning to preprogrammed fluoroscopic views**
Disadvantages	↓Accuracy	↑Cost	↑↑Cost	↑↑↑Cost
	↑Surgeon radiation exposure	↑Time (surgeon- derived registration)		Ergonomics (O-arm is larger than a fluoroscope or Iso-C)

CaIG = computer-assisted image guidance; ↑ Increased ↓ decreased;

* Further studies required to definitively confirm accuracy

** Purported advantages of the O-arm imaging unit

This study represents our initial results of using the O-arm for minimally invasive pedicle screw insertion. The misplacement rate of 7.5% with no breach greater than 2 mm is comparable to previously reported rates of accuracy using CaIG in open spinal procedures. In addition, accurate screw placement was possible even with a significant scoliosis and over four adjacent vertebral segments with a single registration due to the large field of view.

### Study limitations

Although O-arm fluoroscopy with CaIG appears to allow accurate placement of minimally invasive pedicle screws, this study is retrospective and involves a relatively small number of patients. In addition, while all patients underwent a minimally invasive approach in which screws were inserted without direct visualization of the pedicle screw entry sites, the population of patients studied was not uniform, with two patients undergoing a hybrid procedure. To fully validate safety and efficacy, a larger prospective comparative study is required.

## CONCLUSION

The O-arm is a new intraoperative imaging system that is capable of acquiring both fluoroscopic and multi-planar images which can be seamlessly integrated with the StealthStation for CaIG. Minimally invasive pedicle screw insertion utilizing the O-arm and CaIG appears to be safe and accurate.
